# Downregulation of miR-142a Contributes to the Enhanced Anti-Apoptotic Ability of Murine Chronic Myelogenous Leukemia Cells

**DOI:** 10.3389/fonc.2021.718731

**Published:** 2021-07-27

**Authors:** Zhiwei Chen, Yinyin Xie, Dan Liu, Ping Liu, Fei Li, Zhanglin Zhang, Mengmeng Zhang, Xiaolin Wang, Yuanliang Zhang, Xiaojian Sun, Qiuhua Huang

**Affiliations:** ^1^Shanghai Institute of Hematology, State Key Laboratory of Medical Genomics, National Research Center for Translational Medicine at Shanghai, Ruijin Hospital Affiliated to Shanghai Jiao Tong University School of Medicine, Shanghai, China; ^2^Key Laboratory of Systems Biomedicine, Ministry of Education, Shanghai Center for Systems Biomedicine, Shanghai Jiao Tong University, Shanghai, China; ^3^Department of Hematology, The First Affiliated Hospital of Nanchang University, Nanchang, China; ^4^Institute of Hematology, Jiangxi Academy of Clinical Medical Sciences, Nanchang, China; ^5^Department of Transfusion, The First Affiliated Hospital of Nanchang University, Nanchang, China

**Keywords:** chronic myelogenous leukemia, miRNA-mRNA network, apoptosis, miR-142a, Ciapin1

## Abstract

**Background:**

Leukemic stem cell (LSC) is thought to be responsible for chronic myelogenous leukemia (CML) initiation and relapse. However, the inherent regulation of LSCs remains largely obscure. Herein, we integratedly analyzed miRNA and gene expression alterations in bone marrow (BM) Lin^-^Sca1^+^c-Kit^+^ cells (LSKs) of a tet-off inducible CML mouse model, Scl/tTA-BCR/ABL (BA).

**Methods:**

Scl/tTA and TRE-BA transgenic mice were crossed in the presence of doxycycline to get double transgenic mice. Both miRNA and mRNA expression profiles were generated from BM LSKs at 0 and 3 weeks after doxycycline withdrawal. The target genes of differentially expressed miRNAs were predicted, followed by the miRNA-mRNA network construction. *In vitro *and *in vivo* experiments were further performed to elucidate their regulation and function in CML progression.

**Results:**

As a result of the integrated analysis and experimental validation, an anti-apoptotic pathway emerged from the fog. miR-142a was identified to be downregulated by enhanced ERK-phosphorylation in BA-harboring cells, thereby relieving its repression on Ciapin1, an apoptosis inhibitor. Moreover, miR-142a overexpression could partially rescue the abnormal anti-apoptotic phenotype and attenuate CML progression.

**Conclusion:**

Taken together, this study explored the miRNA-mRNA regulatory networks in murine CML LSKs and demonstrated that ERK-miR-142a-Ciapin1 axis played an essential role in CML pathogenesis.

## Introduction

Chronic myelogenous leukemia (CML), a malignant clonal disorder of hematopoietic stem cells (HSCs), is driven by BCR/ABL1 (BA) fusion gene created by translocation t (9; 22)(q34; q11) (1). With the persistent survival, proliferation, and myeloid differentiation advantages, BA-harboring HSCs could generate an overwhelming proportion of myeloid cells in both peripheral blood (PB) and bone marrow (BM) (2). CML is mainly treated by tyrosine kinase inhibitors. However, later-stage CML patients usually suffer from resistance to these inhibitors (3, 4). Hence, the molecular pathogenesis and therapeutic targets of CML still need to be studied comprehensively.

MicroRNAs (miRNA, miR-) are short single-strand, non-coding RNAs with a length of 18-24bp, which was reported for the first time in 1993 (5). miRNAs participate in post-transcriptional regulation by inducing mRNA degradation or repressing mRNA translation. In recent years, several miRNAs were clarified as regulators of hematopoiesis or leukemogenesis. Among them, miR-17-92 is reported to be involved in the regulation of Pu.1-dependent macrophage differentiation (6). miR-146a homozygous knockout mice developed myeloid sarcoma and lymphoma, revealing the tumor suppressor role of miR-146a (7, 8). miR-125b is highly expressed in HSCs, promoting HSC expansion by providing an anti-apoptotic signal (9). miR-142 acts as an oncogene in human T-cell acute lymphoblastic leukemia by accelerating proliferation and inducing glucocorticoid resistance (10). Enforced expression of miR-142-3p could promote myeloid differentiation of AML HSPCs (11).

It has also been reported that miRNAs can be used as biomarkers for CML diagnosis and prognosis. Highly expressed miR-486-5p was detected in CML progenitors. Inhibiting miR-486-5p could reduce the growth of CML progenitors and enhance apoptosis following treatment with imatinib (12). L Venturini et al. demonstrated that BA–c-MYC–miR-17-92 pathway could mediate enhanced miRNA expression in CML CD34^+^ cells at the chronic phase, resulting in the acceleration of disease progression (13). miR-223 expression was shown to be negatively correlated with BA in CML patients, affecting myeloid cell proliferation and transcriptional factor by targeting MEF2C and PTBP2 (14). Downregulation of miR-199b relates to imatinib resistance in CML patients with the deletion of 9q34.1 (15). miR-451 was demonstrated to be inversely associated with BA expression and acted as a predictor of imatinib therapy response in CML (16). miR-142-3p was observed to be upregulated in imatinib-treated K562 (17).

To systematically study the roles of miRNAs in the pathogenesis of CML, herein, we parallelly analyzed BA-induced miRNA and gene expression alterations during CML development using a hematopoietic stem/progenitor cell-specific inducible transgenic murine model, Scl/tTA-BA (18). Based on the target gene prediction and integrated analysis of dysregulated miRNA and mRNA, we built several miRNA-mRNA interaction networks, aiming to elucidate their regulation and function in CML progression.

## Materials and Methods

### Mice Keeping

Transgenic mice used in this study were maintained in the B6 background. Scl/tTA-BA mice were fed with doxycycline (Clontech, USA) in the drinking water (20mg/L) for routine keeping. The animal protocol was reviewed and approved by the Animal Care Committee of Shanghai Jiao Tong University School of Medicine (No. B-2017-010).

### Cell Sorting

BM cells were flushed from tibias and femurs of both legs of each donor into cold DMEM containing 2% FBS and 100ng/mL penicillin/streptomycin. Lineage-positive cells were depleted using the Lineage Cell Depletion Kit (Miltenyi Biotec, Germany). Lineage-negative cells were then stained with Biotin Mouse Lineage Panel (BD Pharmingen, USA), Streptavidin APC-Cy7 (BD Pharmingen, USA), Sca1-PE (BD Pharmingen, USA), and c-Kit-APC (Biolegend, USA) antibody mixture. LSKs were sorted by BD FACSAria (BD Biosciences, USA).

### miRNA microarray and RNA-seq

Total RNA was isolated from BM LSKs using the miRNeasy Mini RNA Isolation Kit (Qiagen, Germany) according to the manual description. RNA integrity number of the total RNA was checked by an Agilent Bioanalyzer 2100 (Agilent Technologies, USA).

For miRNA microarray, miRNAs in total RNA were labeled by miRNA Complete Labeling and Hyb Kit (Agilent Technologies, USA) according to the manufacturer’s instruction. Hybridization was performed using Agilent Mouse miRNA (8*60K) V21.0 microarray. Slides were scanned by Agilent Microarray Scanner (Agilent Technologies, USA) and Feature Extraction software 10.7 (Agilent Technologies, USA) with default settings. Raw data were normalized by the Quantile algorithm that was included in the AgiMicroRna “R” package (19).

For RNA-seq, SMARTer Ultra Low Input RNA for Illumina Kit (Clontech, USA) was used to prepare cDNAs for library construction. Paired-end 100-bp sequencing was performed on the Illumina HiSeq 2500 system (Illumina, USA). The obtained sequence reads (Fastq files) were checked by FastQC software. Clean reads with a minimum length of 25nt were obtained by trimming the raw reads and deleting the low-quality reads, and then the clean reads were mapped to the mouse (GRCm38) genome sequence with 2-bp mismatch using the TopHat software2.0.9 (20). The gene expression levels were estimated by the Fragments Per Kilobase of exon model per Million mapped reads. EdgeR was used for differentially expressed gene analysis (21).

### Bioinformatic Analysis

The gene ontology terms enriched in differentially expressed genes were determined using DAVID bioinformatics resources (22). Gene set enrichment analysis (GSEA) was conducted on RNA-seq data (Broadinstitute, Cambridge, USA) (23). Gene set files were generated according to fingerprint genes of specific hematopoietic cells that were reported by Stuart M. Chambers et al. (24). The miRNA target genes were predicted by the miRwalk2.0 database using a newly developed algorithm named ‘miRWalk’ as well as with already established programs for putative miRNA binding sites (25).

### Stable Cell Line Generation

32Dcl3 (32D) cells were infected separately by pMSCV-BA-IRES-mCherry and pMSCV-IRES-mCherry retrovirus supernatants in the culture medium supplemented with 8μg/mL polybrene (Sigma-Aldrich, USA). mCherry**^+^** cells were isolated by flow cytometry after 48 hours and sorted again after one week to generate the stable cell line expressing BA-IRES-mCherry (32D-BA) or mCherry alone (32D-mCherry). 32D-BA cells were infected separately by pMSCV-Ciapin1-IRES-puro, pMSCV-IRES-puro, pLKO-sh-Ciapin1, and pLKO-scramble virus supernatants, after 48 hours, 1μ**g**/ml puromycin (Beyotime, China) was added in culture medium for 3 days to select the stable cell line expressing Ciapin1 (32D-BA-Ciapin1), puro alone (32D-BA-puro), Ciapin1 knockdown (BA-sh-Ciapin1), and scramble alone (BA-sh-scramble). 32D and 32D-mCherry cells were maintained in RPMI 1640 medium (Gibco, China) supplemented with 10% fetal bovine serum (Gibco, Australia) and 10% IL-3 containing WEHI-3B conditional medium. 32D-BA, 32D-BA-Ciapin1, 32D-BA-puro, BA-sh-Ciapin1, and BA-sh-scramble cells were maintained in RPMI 1640 supplemented with 10% fetal bovine serum.

### Quantitative Reverse Transcription PCR (qRT-PCR)

Total RNA was isolated with TRIzol reagent and further purified with the miRNeasy Micro Kit (Qiagen, Germany) according to the manufacturer’s instruction. For miRNA qRT-PCR, Mir-X™ miRNA First-Strand Synthesis Kit (Clontech, USA) was used to generate cDNAs. The entire sequence of mature miRNA (21–23nt) was used as a miRNA-specific 5’primer. The mRQ 3’Primer was supplied in the Kit. The Delta-delta Ct method was used to determine the level of each miRNA relative to the level of sno202 snRNA.

For qRT-PCR, cDNAs were synthesized using the PrimeScript™ RT Reagent Kit with gDNA Eraser Kit (Takara, Japan). The gene expression level was determined by SYBR Green II qPCR. Rn18s was used as an internal control. The following primer-pairs were used for the target gene amplification: Rn18s forward primer 5’ GCAATTATTCC CCATGAACG 3’ and reverse primer 5’ GGCCTCACTAAAC-CATCCAA 3’; Bcl2 forward primer 5’ ATGCCTTTGTGGAA CTATATGGC 3’ and reverse primer 5’ GGTATGCACCCAGA GTGATGC 3’; Birc2 forward primer 5’ TGTGGCCTGATGTTG GATAAC 3’ and reverse primer 5’ GGTGACGAATGTG-CAAATCTACT 3’; Ciapin1 forward primer 5’ GGAGTTTG-GGATCTCCCCTG 3’ and reverse primer 5’ ACCCGACA GAATGACATCGAA 3’; Xiap forward primer 5’ CGAGCTGG GTTTCTTTATACCG 3’ and reverse primer 5’ GCAATTTG GGGA-TATTCTCCTGT 3’.

### Cell Viability and Apoptosis Assay

miRNA mimics and the negative control (NC) oligonucleotides were synthesized by Shanghai GenePharma Co., Ltd (Shanghai, China). The viability of 32D-BA cells was assayed using the CCK-8 Kit (Dojindo, Japan). Cells were plated in triplicate at a density of 1×10**^4^**/well in a 96-well plate at 24h hours after transfection with miRNA mimics. CCK-8 reagent (10μL) was added to each well at 24, 48, and 72h, respectively. The optical density at 450nm was measured after one hour of incubation. Apoptosis ability of murine BM cells was assessed using annexin V/PI (BD Pharmingen, USA) staining.

### Luciferase Reporter Assay

3´untranslated region (3´UTR) of the target gene was inserted into the pmirGlo vector, and an empty vector was used as the control. Murine pre-miR-142a sequence, ~100nt of 5’- and 3’-flanking region were amplified from genomic locus by PCR and cloned into pMSCV-IRES-mCherry vector. Transient transfection was performed in 293T cells with 0.8μg pMSCV- miR142a-IRES-mCherry plasmid and 0.2 μg pmirGlo-control or pmirGlo-3’UTR reporter construct. The fluorescent activity was measured consecutively using Dual-Luciferase assays (Promega, UK) 48 hours after transfection, according to the instructions of the manufacturer.

### Western Blot Analysis

Cells were counted and lysed in 1×SDS running buffer, heated at 100°C for 10 minutes and centrifuged to remove debris. Protein samples were separated on polyacrylamide gels, transferred onto a PVDF membrane and then blotted with the following primary antibodies: anti-β-actin antibody (Proteintech, China), anti-Bcl-2 antibody (Cell Signaling Technology, USA), anti-Birc2 antibody (Cell Signaling Technology, USA), anti-Ciapin1 antibody (Proteintech, China), anti-Erk1/2 antibody (Cell Signaling Technology, USA), anti-Phospho-Erk1/2 (Cell Signaling Technology, USA), anti-Pu.1 antibody (Cell Signaling Technology, USA), and anti-Xiap antibody (Cell Signaling Technology, USA). HRP-labeled goat anti-mouse IgG (Proteintech, China) or goat anti-rabbit IgG (Proteintech, China) was used as a secondary antibody.

### Viral Vectors Construction and Infection

293T cells were transfected with the pCDH-EF1-miR142a-T2A-copGFP plasmid accompanied by pMD2.G and psPAX2, or pMSCV-BA-IRES-mCherry plasmid accompanied by EcoPack using Lipofectamine 2000 (Invitrogen, USA) as per the manufacturer’s protocol. Viral supernatant was collected 48 hours after transfection and concentrated by centrifugation.

### BM Transplantation

Balb/c donor mice were treated with 5-fluorouracil 5 days before harvest of BM from tibias and femurs. BM cells were infected by the pCDH-EF1-miR142a-T2A-copGFP and pMSCV-BA-IRES-mCherry virus twice. 1.1×10**^6^** total infected cells were resuspended in 200μl PBS and injected into the tail vein of each lethally irradiated female Balb/c recipient (6-8 weeks old). PB cells were analyzed for the expression level of mCherry, Mac1, and Gr1, as well as the leukocyte number two weeks later.

### Statistical Analysis

Graphpad Prism software was used to create graphs and perform statistical analyses. The significance of differences in results, including apoptosis rates, mRNA levels, WBC counts, and percentages of mCherry^+^ cells in PB after BMT was calculated by the student’s unpaired t-test.

## Nomenclature

32Dcl23 cell line: RRID: CVCL_6G54
Doxycycline: Clontech Cat# 631311
Lineage Cell Depletion Kit, mouse: Miltenyi Biotec Cat# 130-090-858
Biotin Mouse Lineage Panel: BD Pharmingen™ Cat# 559971
Streptavidin APC-Cy™7: BD Pharmingen™ Cat# 554063
Sca-1 PE: BD Biosciences Cat# 561076, RRID: AB_2034020
CD117 (c-kit)-APC: BioLegend Cat# 135108
miRNeasy Mini Kit: QIAGEN Cat# 217004
Mir-X™ miRNA First-Strand Synthesis Kit: Clontech# 638313
PrimeScript™ RT Reagent Kit with gDNA Eraser Kit: Takara# RR047A
Cell Counting Kit-8: Dojindo# CK04-05
Dual-Luciferase assays: Promega# E1910
β-Actin antibody (C4): Proteintech Cat# 60008-1-Ig, RRID: AB_2289225
Bcl-2 (124) antibody: Cell Signaling Technology Cat# 15071, RRID : AB_2744528
Birc-2 antibody: Cell Signaling Technology Cat# 4952, RRID : AB_2063660
CIAPIN1 antibody: Proteintech Cat# 12638-1-AP, RRID : AB_2079665
Pu.1 antibody: Cell Signaling Technology Cat #2258, RRID : AB_2186909
Xiap antibody: Cell Signaling Technology Cat# 2042, RRID : AB_2214870

## Results

### Induced Expression of BA Altered miRNA and Gene Profiles of Murine BM LSKs

**Figure 1:**
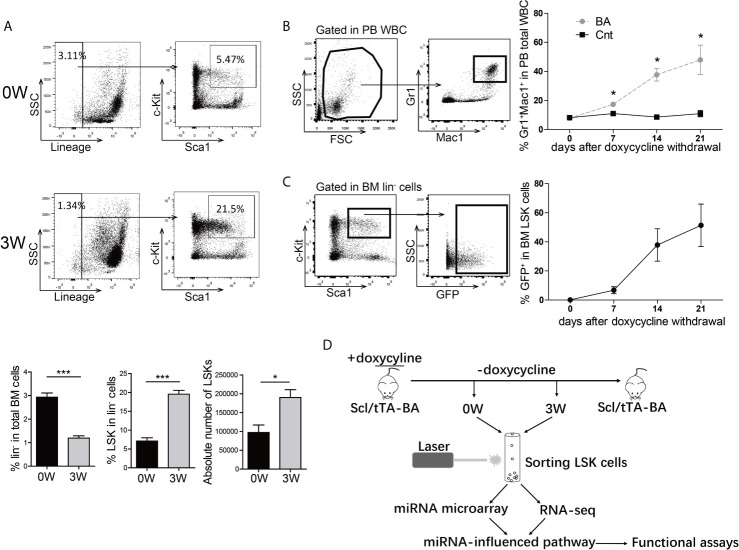
BCR/ABL1 (BA) interferes with the proportion of LSKs in murine bone marrow (BM). **(A)** Proportion and absolute number analysis of BM Lin^-^Sca-1^+^c-Kit^+^ (LSK) cells in Scl/tTA-BA mice before and after doxycycline withdrawal. **(B)** Follow-up analysis of granulocyte proportion in peripheral blood (PB) of Scl/tTA-BA mice after doxycycline withdrawal. **(C)** Follow-up analysis of GFP^+^ cell proportion in LSKs of Scl/tTA-GFP mice. **(D)** Schematic design of the experiment. Results are mean ± standard error of the mean (SEM), *p < 0.05, ***p < 0.001 **(A, B)** was assessed by unpaired *t*-test.

To get insight into the inherent regulation of CML LSCs, a tet-off inducible transgenic murine CML model, Scl-tTA-BA (18), was chosen for integrated analysis of the miRNA-mRNA regulatory network in BM LSKs. All mice developed a CML-like disease after doxycycline withdrawal (**Figure S1**). As CML is a stem cell-derived disease, the number and proportion of BM HSPC were analyzed three weeks after doxycycline withdrawal. The proportion and the absolute number of BM LSKs significantly increased in BA-harboring mice compared with that in the control group (**Figure 1A**). To choose the appropriate point of time for subsequent analysis, the percentage of PB granulocytes was followed up after doxycycline withdrawal, showing that the granulocyte percentage rose from 8% at week-0 to 50% at week-3 (**Figure 1B**). Furthermore, parallel detection of GFP^+^ proportion in BM LSKs of Scl/tTA-GFP mice was performed as a reference for the evaluation of BA protein level (26). At week-0, there were no GFP^+^ cells in LSKs, while almost half of the LSKs were GFP^+^ at week-3 (**Figure 1C**). Therefore, we sorted BM LSKs at week-0 and week-3 after doxycycline withdrawal and extracted RNA for subsequent miRNA microarray and RNA-seq experiments (**Figure 1D**).

**Figure 2:**
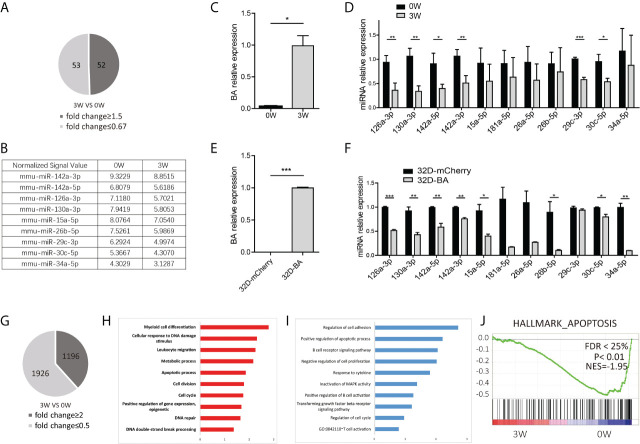
miRNA and mRNA alteration of BM LSKs in Scl/tTA-BA mice. **(A)** Number of dysregulated miRNAs detected by miRNA array (fold-change ≥1.5 or ≤0.67). **(B)** Normalized signal values of representative differentially expressed miRNAs. **(C)** Expression detection of BA in BM LSKs of Scl/tTA-BA mice. **(D)** qRT-PCR confirmation of expression level of selected miRNAs in BM LSKs from Scl/tTA-BA mice. **(E)** Expression detection of BA in 32D-BA cell line. **(F)** qRT-PCR confirmation of expression level of selected miRNAs in 32D-BA cell line. **(G)** The number of dysregulated genes detected by RNA-seq (fold-change≥2 or ≤0.5). **(H, I)** GO analysis of differentially expressed genes between BA-harboring and normal BM LSKs. **(J)** GSEA showing that the apoptosis pathway was remarkably downregulated in BM LSKs at week-3 after doxycycline withdrawal. Results are mean ± SEM, *p < 0.05, **p < 0.01, ***p < 0.001 **(C–F)** was assessed by unpaired *t*-test.

Compared with mice fed on doxycycline (week-0), there are 52 miRNAs upregulated with fold change≥1.5 and 53 miRNAs downregulated with fold change ≤ 0.67 at week-3 (**Figures S2** and **2A**). The dysregulated miRNAs are listed in **Supplementary Table S1** and **Figure 2B**. Some of them, such as miRNA-26a-5p, miR-30c-5p, miR-142a-5p, miR-142a-3p, and miR-126a-3p, miR-130a-3p, and miR-34a-5p, have been known to play important roles in solid cancer or leukemia development (27–34). To further validate the array data, qRT-PCR was used to detect the expression level of BA and several selected downregulated miRNAs in LSK samples. As expected, BA expression level was significantly upregulated at week-3 after doxycycline withdrawal (**Figure 2C**). For miRNAs, consistent with array data, miR-126a-3p, miR-130a-3p, miR-142a-5p, miR-142a-3p, miR-29c-3p, and miR-30c-5p decreased significantly following BA expression. Another three miRNAs also had a decreasing tendency, though showed no statistical difference compared with that in the un-withdrawal sample (**Figure 2D**). Besides, a murine IL-3-dependent myeloid cell line, 32D, was also used to validate array data. Stable cell lines 32D-BA and 32D-mCherry were constructed (**Figure 2E**). Enforced expression of BA resulted in significant downregulation of most selected miRNAs (**Figure 2F**). Overall, our results demonstrated that induced expression of BA altered miRNA profiles of murine BM LSKs.

Meanwhile, global gene expression analysis was performed on BM LSKs of Scl-tTA-BA mice (**Figure S3**). LSKs were also collected at week-0 or week-3 after doxycycline withdrawal. After data preprocessing, we identified 3122 differentially expressed genes that contain 1196 upregulated and 1926 downregulated genes (**Figure 2G**). The top 100 dysregulated mRNAs are listed in **Supplementary Table S2**. GO analysis indicated that upregulated genes are involved in a broad range of biological process functions, including myeloid cell differentiation, cellular response to DNA damage stimulus, leukocyte migration, metabolic process, apoptosis, and cell cycle etc. (**Figure 2H**). Downregulated genes were mainly enriched in the regulation of cell adhesion, positive regulation of the apoptotic process, and negative regulation of cell proliferation etc. (**Figure 2I**). In line with this, GSEA on a customized gene set file, a gene expression fingerprint of specific hematopoietic cells from Chambers et al. (24), also showed significant depletion of the apoptosis signature in LSKs harbored BA fusion gene (**Figure 2J**).

### Integrative Analysis of miRNA−mRNA Network Revealed an Enhanced Cell Survival Probability of CML LSKs

**Figure 3:**
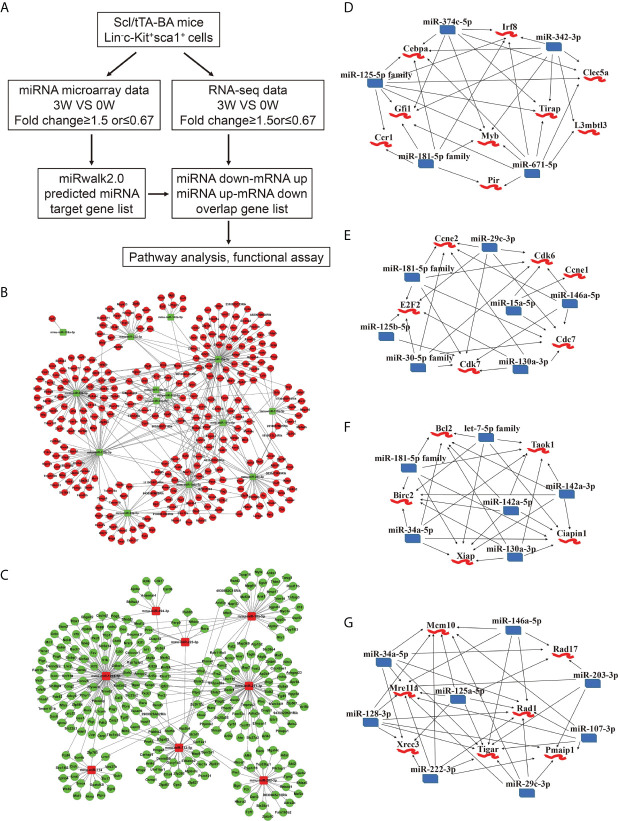
Integrated analysis of miRNA-mRNA regulatory networks in murine CML LSKs. **(A)** Integrated analysis flowchart of the screened miRNAs and predicted target genes. **(B, C)** The correlation networks showing 431 interactions built between 14 downregulated miRNAs and 299 possible target genes **(B)**, and 318 interactions between 8 upregulated miRNAs and 271 possible target genes **(C)**. **(D–G)** Visualization of the regulatory networks enriched for four pathways including myeloid differentiation **(D)**, cell cycle **(E)**, anti-apoptotic pathway **(F)**, and DNA damage **(G)**.

Next, basing on the data above, we performed an integrated analysis of dysregulated miRNAs and genes. First, target genes of miRNAs were predicted using miRwalk2.0 database. Second, we overlapped the predicted target genes with dysregulated genes detected by RNA-seq and proposed a regulatory network of miRNA-mRNA (**Figure 3A**). 431 interactions were built between 14 downregulated miRNAs and 299 upregulated genes (**Figure 3B**). The edges that connect nodes showed the interaction of miRNAs and mRNAs, and the nodes represent miRNAs or genes, red indicating upregulation, and green indicating downregulation. We also built 318 interactions between 8 upregulated miRNAs and 271 downregulated mRNAs (**Figure 3C**). Among the dysregulated ones shown in **Figures 3B, C**, several miRNAs and their target genes were known to be involved in CML progression (35–38).

As reported, CML progression can be promoted by multiple pathways including RAS, PI3K/AKT, ERK, MYC, JAK/STAT, myeloid differentiation, apoptosis, cell cycle, and genomic instability (39). In the light of the information uncovered by integrated analysis, we especially focused our attention on four pathways, including anti-apoptotic, cell cycle, myeloid differentiation, and DNA damage pathway. A regulatory network emerged, gathering miR-374c-5p, miR-342-3p, miR-671-5p, miR-181-5p family, and miR-125-5p family. All these miRNAs are downregulated following BA expression. *Cebpa, Gfi1, Myb, Irf8*, and *Tirap* etc., whose upregulation has been reported to regulate myeloid differentiation positively, are involved in this network (**Figure 3D**). Furthermore, the interactions between downregulated miR-181-5p family, miR-29c-3p, miR-15a-5p, miR-146a-5p, miR-130a-3p, miR-125b-5p, and miR-30-5p family and upregulated genes Ccne1, Ccne2, Cdk6, Cdk7, Cdc7, and E2F2 were identified. The predicted target genes in this network are all involved in the cell cycle (**Figure 3E**). miR-130a-3p, miR-142a-5p, miR-142a-3p, miR-34a-5p, miR-181-5p family, let-7-5p family, and highly expressed genes, including Bcl2, Birc2, Ciapin1, Xiap, and Taok1, were weaved into a network related to the anti-apoptotic process, which implicated that BA expression could promote cell survival (**Figure 3F**). Last but not least, we displayed a network of several downregulated miRNAs (miR-146a-5p, miR-203-3p, miR-107-3p, miR-29c-3p, miR-222-3p, miR-125a-5p, miR-128-3p, and miR-34a-5p) and their predicted target genes (Mcm10, Rad17, Rad1, Pmaip1, Tigar, Mre11a, and Xrcc3), which is involved in the cellular response to DNA damage stimulus (**Figure 3G**).

**Figure 4:**
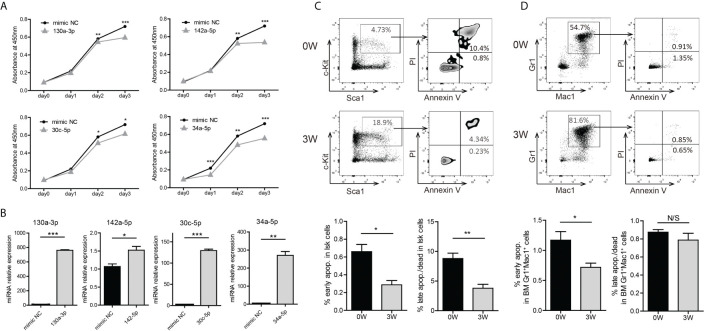
Induction of BA expression enhanced cell survival probability of murine BM LSKs. **(A)** Cell viability analysis of 32D-BA cells transfected with selected miRNAs by CCK8 assays. **(B)** Validation of transfection efficiency of miRNAs by qRT-PCR experiments. **(C, D)** Apoptosis analysis of BM LSKs **(C)** and granulocytes **(D)** *via* Annexin V/PI staining (n=3). Results are mean ± SEM, *p < 0.05, **p < 0.01, ***p < 0.001 and N/S (not significant) **(A–D)** was assessed by unpaired *t*-test.

To evaluate the biologic function of these miRNAs in BA-expressing cells, we transfected some of the mimics into 32D-BA cells and performed a CCK-8 assay for cell viability analysis. The viability of 32D-BA cells could be suppressed by miR-130a-3p, miR-142a-5p, miR-30c-5p, and miR-34a-5p (**Figure 4A**). The transfection efficiency of these mimics was determined by qRT-PCR analysis (**Figure 4B**). These data indicated that the downregulation of these miRNAs might promote cell survival. Apoptosis analysis was then performed on BM cells collected from Scl-tTA-BA mice. A significant decrease in the proportion of apoptotic cells in LSKs was detected in both early and late apoptosis at week-3 after doxycycline withdrawal compared with that at week-0 (**Figure 4C**). Besides, we observed that the early apoptosis was suppressed in granulocytes following BA expression, while the late apoptosis was not disturbed (**Figure 4D**).

### Downregulation of miR-142a Promoted the Survival of BA-Harboring Cells by Relieving Its Repression on Ciapin1

**Figure 5:**
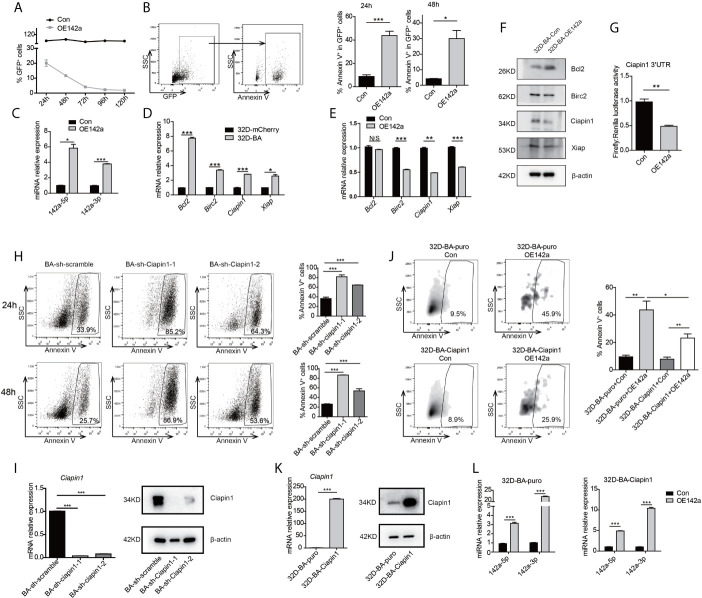
Overexpression of miR-142a impaired cell viability partially through targeting Ciapin1. **(A)** Follow-up analysis of GFP^+^ cell proportion in 32D-BA cells infected with pCDH-EF1-miR142a-T2A-copGFP virus. **(B)** Apoptosis analysis of 32D-BA cells at 24h and 48h after lentivirus infection. **(C)** Validation of miR-142a-5p and miR-142a-3p expression by qRT-PCR. **(D, E)** qRT-PCR detection of anti-apoptotic gene Bcl2, Birc2, Ciapin1, and Xiap in 32D-BA cells **(D)** and miR-142a-overexpressed (OE142a) 32D-BA cells **(E)**. **(F)** Western blot detection of anti-apoptotic proteins Bcl2, Birc2, Ciapin1, and Xiap in 32D-BA cells infected with lentivirus encoding miR-142a. **(G)** Identification of miR-142a target gene by luciferase reporter assay. **(H)** Apoptosis analysis of 32D-BA cells at 24h and 48h after Ciapin1 knockdown. **(I)** Determination of Ciapin1 expression by qRT-PCR and western blot analysis. **(J)** Comparative analysis of apoptosis between 32D-BA-puro+OE142a and 32D-BA-Ciapin1+OE142a cells *via* Annexin V staining. **(K)** Validation of Ciapin1 expression by qRT-PCR and Western blot assay. **(L)** Validation of miR-142a-5p and miR-142a-3p expression in 32D-BA-puro and 32D-BA-Ciapin1 cells by qRT-PCR. Results are mean ± SEM, *p < 0.05, **p < 0.01, ***p < 0.001 and N/S (not significant) **(B–E, G–L)** was assessed by unpaired *t*-test.

As both miR-142a-5p and miR-142a-3p were involved in a network related to the anti-apoptotic process and detected to be downregulated in CML LSKs and 32D-BA cells (**Figures 2D, F**), we then conducted enforced overexpression of miR-142a (OE142a) in 32D-BA cells by infection with pCDH-EF1 lentivirus encoding miR-142a-T2A-GFP. Our results showed that miR-142a overexpression could gradually reduce the survival ability of 32D-BA cells (**Figure 5A**) and significantly promoted cell apoptosis (**Figure 5B**). The overexpression levels of miR-142a-5p and miR-142a-3p were confirmed by qRT-PCR (**Figure 5C**). These results suggested that miR-142a overexpression induced cell apoptosis in BA-harboring cells.

In line with our prediction of anti-correlated miRNA-target gene pairs, the mRNA level of anti-apoptotic genes Bcl2, Birc2, Ciapin1, and Xiap were observed to be upregulated in 32D-BA cells but downregulated by miR-142a overexpression (**Figures 5D, E**). Of note, the protein level of Ciapin1 was also found to be downregulated by miR-142a overexpression (**Figure 5F**). Furthermore, luciferase reporter assays revealed that Ciapin1 might be the direct target of miR-142a (**Figure 5G**). To verify the involvement of Ciapin1 in the miR-142a-mediated cellular effect, we treated 32D-BA cells with shRNA-Ciapin1 and detected the cell apoptosis ratio. As expected, a significant increase in apoptosis was observed (**Figures 5H, I**). Next, we co-overexpressed Ciapin1 and miR-142a in 32D-BA cells, finding that Ciapin1 could partially rescue the impaired cell survival ability induced by miR-142a (**Figure 5J**). The overexpression levels of Ciapin1 and miR-142a were shown in **Figures 5K, L**. Taken together, these findings suggest that the downregulation of miR-142a contributes to the enhanced anti-apoptotic ability of BA-harboring cells by relieving its repression on Ciapin1.

### Phosphorylated ERK Inhibits the Expression of miR-142a in BA-Harboring Cells

Previous studies have shown that Pu.1 was critical for miR-142 gene expression in murine dendritic cells, and PU.1 expression was severely impaired in CML patients (40, 41). It’s of interest whether Pu.1 was critical for miR-142 gene expression in BA-harboring cells. To verify this point, we detected the expression level of Pu.1 in 32D-BA cells, showing only a slight decrease of Pu.1 mRNA level as compared with that in 32D-mCherry cells (**Figure S4A**). Subsequently, 32D-BA cells were transducted with flag-Pu.1-expressing retroviral supernatants (**Figure S4B**). Pu.1 overexpression induced a slight increase in miR-142a-5p expression but no significant difference in miR-142a-3p expression (**Figure S4C**). These data indicated that Pu.1 might not be the critical reason for miR-142a downregulation in BA-harboring cells.

**Figure 6:**
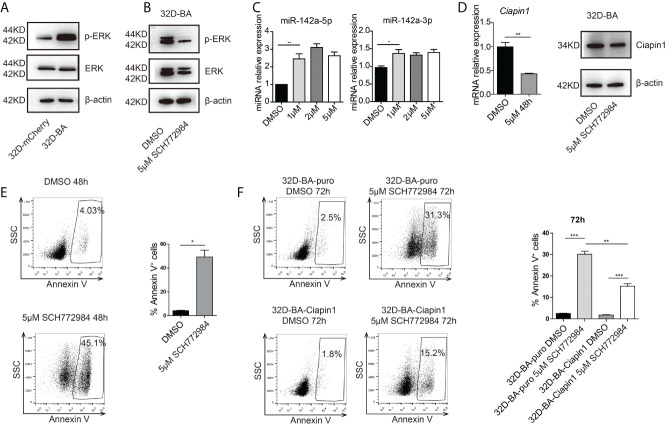
Phosphorylated (P) ERK inhibited the expression of miR-142a in BA-harboring cells. **(A)** Western blot detection of total and p-ERK protein level in 32D-BA and 32D-mCherry cells. **(B)** The ERK inhibitor SCH772984 inhibited the p-ERK level in 32D-BA cell line. **(C)** qRT-PCR detection of miR-142a-5p and miR-142a-3p level in 32D-BA cells with ERK inhibitor treatment. **(D)** qRT-PCR and Western blot analysis of Ciapin1 expression in 32D-BA cells with ERK inhibitor treatment. **(E)** Apoptosis analysis of 32D-BA cells with ERK inhibitor treatment. **(F)** Apoptosis analysis of 32D-BA-Ciapin1 cells with ERK inhibitor treatment. Results are mean ± SEM, *p < 0.05, **p < 0.01, ***p < 0.001 **(C–F)** was assessed by unpaired *t*-test.

Another study has reported the bimodal regulation of miR-142a by the ERK pathway in mice embryonic stem cells (42). Does ERK play a pivotal role in the downregulation of miR-142a in BA-harboring cells? To address this point, we checked the expression level of ERK in 32D-BA cells. Highly phosphorylated ERK was showed in 32D-BA cells when comparing with 32D-mCherry cells (**Figure 6A**). Next, we treated 32D-BA cells with an ERK inhibitor (SCH772984) for 24 hours, a significant decrease of ERK phosphorylation was observed as compared with DMSO treated group (**Figure 6B**). Meanwhile, both miR-142a-5p and miR-142a-3p were upregulated with the treatment of different dosages (1μM, 2μM, and 5μM) (**Figure 6C**). Moreover, the mRNA level of Ciapin1, as well as the protein level, was repressed in ERK inhibitor-treated 32D-BA cells (**Figure 6D**), along with the significant increase of apoptosis rate (**Figure 6E**). Lastly, we treated stable 32D-BA-Ciapin1 cells with 5μM SCH772984 to examine whether Ciapin1 overexpression can rescue the impaired cell survival ability induced by ERK inhibitor in 32D-BA cells. Indeed, a significant decrease in apoptosis was observed at 72h after treatment (**Figure 6F**). These results supported that miR-142a was partially downregulated by BA-triggered ERK phosphorylation and then promoted leukemia cell survival through relieving its repression on Ciapin1.

### miR-142a May Act As a Suppressor in CML Progression

**Figure 7:**
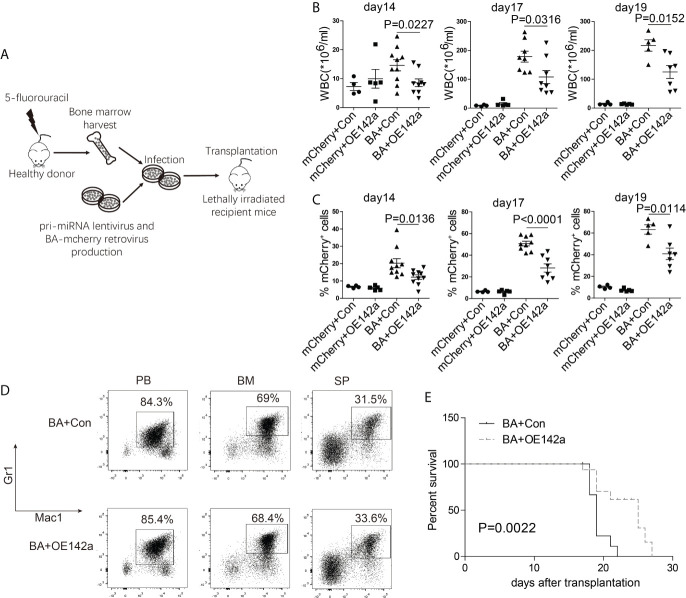
miR-142a may act as a suppressor in CML progression. **(A)** Schematic of miR-142a treatment on BA-induced CML mice. **(B)** Follow-up analysis of the PB white blood cell (WBC) count after transplantation. **(C)** Follow-up analysis of the proportion of mCherry^+^ cells in PB. **(D)** Immunophenotype analysis of mice in BA and BA+miR-142a group. **(E)** Kaplan-Meier survival curve of mice inoculated with miR-142a overexpression or control lentivirus. Results are mean ± SEM, p value was assessed by unpaired *t*-test.

Next, we assessed the effect of miR-142a overexpression on CML progression *in vivo*. BM cells from 5-fluorouracil treated healthy Balb/c mice were collected and co-infected with the pCDH-EF1-miR142a-T2A-copGFP and pMSCV-BA-IRES-mCherry viral supernatant, then transplanted into lethally irradiated wild-type Balb/c recipients (**Figure 7A**). The pCDH-EF1-T2A-copGFP and pMSCV-IRES-mCherry virus were used as empty controls. We then closely monitored the WBC count and the percentage of mCherry^+^ (BA positive) cells in PB. As our results showed, miR-142a overexpression reduced the WBC count (**Figure 7B**) as well as the ratio of BA positive cells in recipients (**Figure 7C**). Though Mice in both BA+OE142a group and BA+control group showed a CML-like phenotype (**Figure 7D**), overexpression of miR-142a significantly prolonged the survival of mice (**Figure 7E**). These results suggest that miR-142a may act as a suppressor in CML progression.

## Discussion

In this study, we parallelly analyzed BA-induced miRNA and gene alterations to systematically study the roles of miRNAs in the pathogenesis of CML by using a hematopoietic stem/progenitor cell-specific tet-off inducible transgenic murine model, Scl/tTA-BA, in which doxycycline withdrawal can induce BA expression in HSPCs, leading to a CML-like phenotype. This model provides an incomparable advantage for studying CML pathogenesis.

On the basis of the target gene prediction and integrated analysis of dysregulated miRNA and mRNA, we built several miRNA-mRNA interaction networks, including the apoptosis process, cell cycle, myeloid differentiation, and DNA damage. Interestingly, a group of differentially displayed genes in CML LSCs (CD34^+^/CD38^-^) and CML progenitors (CD34^+^/CD38^+^) generated from CML patients also appeared to be involved in these four processes (43). As shown in the result, miR-130a-3p, miR-142a-5p, miR-142a-3p, miR-34a-5p, miR-181-5p family, and let-7-5p family were weaved into the apoptosis network. Subsequent experiments further confirmed that both early and late apoptosis of BM LSKs in BA-induced mice were decreased compared with the control mice (**Figure 4C**). In line with this, miR-181a has been reported to induce apoptosis in CML (44), and miR-34a was found to be associated with apoptosis regulation in solid tumours (45, 46).

According to the information uncovered by integrated analysis of our miRNA and mRNA-seq data, miR-142a was negatively correlated with an apoptosis inhibitor, Ciapin1, which much interested us. miR-142 is specifically expressed in hematopoietic cells and dynamically regulated during hematopoiesis commitment (47); the expressions of miR-142-3p and miR-142-5p have been known to be positively correlated with the degree of cell differentiation (48, 49). Usually, miR-142 dysregulation is accompanied by hematopoiesis development disorder and leukemia genesis. It is thought to be an oncogene for its capability to accelerate proliferation and induce glucocorticoid resistance in human T-cell acute lymphoblastic leukemia (10). Enforced expression of miR-142-3p will promote myeloid differentiation of HSPCs from AML patients (11). The upregulation of miR-142-3p is positively correlated with drug sensitivity in AML (50). However, the expression level of miR-142 in CML is inconclusive. Letizia Venturini’s data showed that in several cases, miR-142-3p was downregulated, while miR-142-5p was upregulated in CD34^+^ cells (11). miR-142-3p was also observed to be upregulated in imatinib-treated K562 (17). However, in other data (GSE28825), both miR-142-5p and miR-142-3p showed a downward trend after imatinib treatment on K562 (51). Our data showed that both miR-142a-5p and miR-142a-3p were downregulated after doxycycline withdrawal in BM LSKs of BA-induced CML mice, which may partially take responsibility for the pro-survival effect on BA-harboring cells. Indeed, we observed that enforced overexpression of miR-142a could induce apoptosis of 32D-BA cells and prolong the survival of BA-induced CML mice. Our results suggested that miR-142a might act as a suppressor in CML progression.

miR-142 downregulation has been found in multiple diseases with diverse mechanisms. In mouse dendritic cells, Pu.1, Runx1, and C/EBPβ constitutively occupy miR-142 promoter region (40). The expression level of PU.1 in chronic phase CML patients was downregulated, and it could be rescued by treatment with interferon-alpha or imatinib (41). On the basis of this, we were interested in whether Pu.1 was critical for miR-142a expression in BA-expressing cells. However, our result showed that enforced overexpression of Pu.1 in 32D-BA cells couldn’t rescue the abnormal miR-142a expression induced by BA.

Another study reported that inhibiting the ERK pathway can upregulate miR-142a expression in mice embryonic stem cells (42). Treatment with MAPK inhibitor compounds could block the downregulation of both miR-142-5p and miR-142-3p in cardiac myocytes (52). Consistent with this, we identified that both miR-142a-5p and miR-142a-3p were upregulated in 32D-BA cells following the treatment with ERK inhibitor (**Figure 6C**). Along with this, cell apoptosis was also increased. These data indicated that miR-142a expression was partially inhibited by highly phosphorylated and activated ERK in BA-harboring cells.

Epigenetic modifications are also involved in the regulation of miR-142 expression. In human nasopharyngeal carcinoma, EZH2 recruits DNA methyltransferase DNMT1 and induces hypermethylation in the promoter region of miR-142, thereby inhibiting its expression (53). In Systemic lupus erythematosus CD4^+^ T cells, the miR-142 expression is downregulated by BCL-6 through modulating histone methylation and acetylation on the promoter of miR-142 (54). But whether these regulation mechanisms exist in BA-harboring cells is unclear, which may require further investigation.

Overall, this study is the first to systematically identify the miRNA-mRNA regulation network of LSKs in an inducible CML model. Our results showed that the ERK-miR-142a-Ciapin1 axis partially prevented BA-harboring cells from apoptosis. However, whether the integrated regulatory axis exists in CML patient samples, especially in the CD34^+^ subpopulation, remains unknown. It will be an important point for us in the next step.

## Conclusion

Collectively, this study explored the miRNA-mRNA regulatory networks of BM LSKs in an inducible CML murine model and suggested that the ERK-miR-142a-Ciapin1 axis might play an essential role in enhancing the anti-apoptotic ability of CML cells.

## Data Availability Statement

The original contributions presented in the study are included in the article/**Supplementary Material**. Further inquiries can be directed to the corresponding author.

## Ethics Statement

The animal study was reviewed and approved by The Animal Care Committee of Shanghai Jiao Tong University School of Medicine (No. B-2017-010).

## Author Contributions

ZC and YX equally contributed to the design of the study, the performance of the experiments. DL, PL, FL, ZZ, MZ, and XW performed the experiments. ZC, YX, YZ, XS, and QH analyzed the data. ZC and QH wrote the manuscript. All authors contributed to the article and approved the submitted version.

## Funding

This work was supported by the National Natural Science Foundation of China (81900149), Shanghai Municipal Education Commission-Gaofeng Clinical Medicine Grant Support (20152506), and Samuel Waxman Cancer Research Foundation.

## Conflict of Interest

The authors declare that the research was conducted in the absence of any commercial or financial relationships that could be construed as a potential conflict of interest.

## Publisher’s Note

All claims expressed in this article are solely those of the authors and do not necessarily represent those of their affiliated organizations, or those of the publisher, the editors and the reviewers. Any product that may be evaluated in this article, or claim that may be made by its manufacturer, is not guaranteed or endorsed by the publisher.
