# Supplementary material for: Downregulation of miR-142a Contributes to the Enhanced Anti-Apoptotic Ability of Murine Chronic Myelogenous Leukemia Cells
Source: Front Oncol. 2021 Jul 27;11:718731. doi: 10.3389/fonc.2021.718731 (PMC8354203; doi:10.3389/fonc.2021.718731)
Supplement: Supplementary file 1 [file DataSheet_1.docx]

**
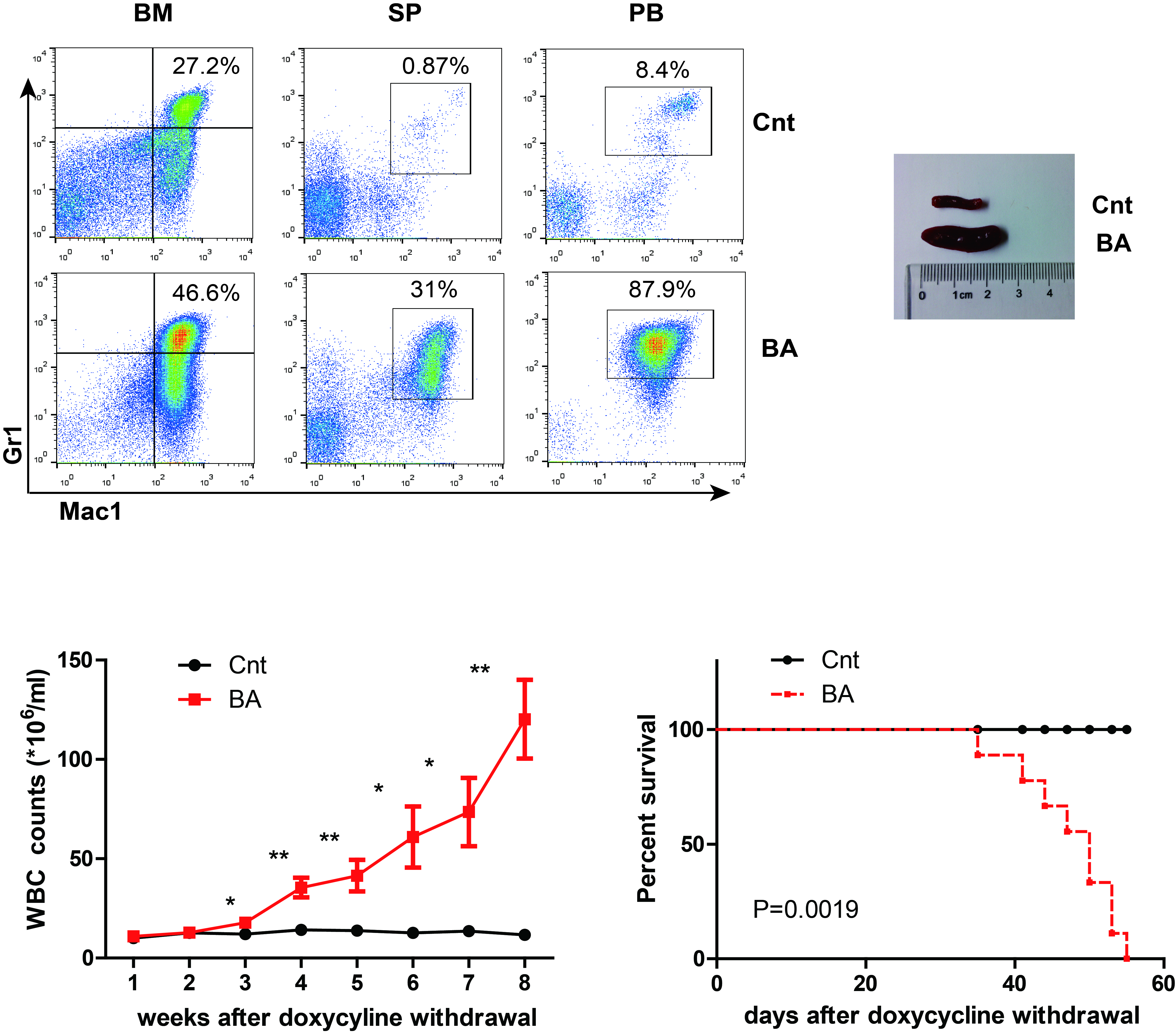
**

D

C

B

A

**Figure S1. CML-like phenotype in Tet-off inducible Scl/tTA-BCR/ABL (Scl/tTA-BA) transgenic mice.** **(A)** Proportion analysis of granulocytes in BM, SP, and PB of WT and moribund Scl/tTA-BA mice by flow cytometry. **(B)** Spleen size in WT and moribund Scl/tTA-BA mice. **(C)** 8 week follow-up of WBC count after doxycycline withdrawal. **(D)** Survival curve analysis after doxycycline withdrawal. Results are expressed as mean ± SEM, p value was assessed by unpaired t-test at each time point.


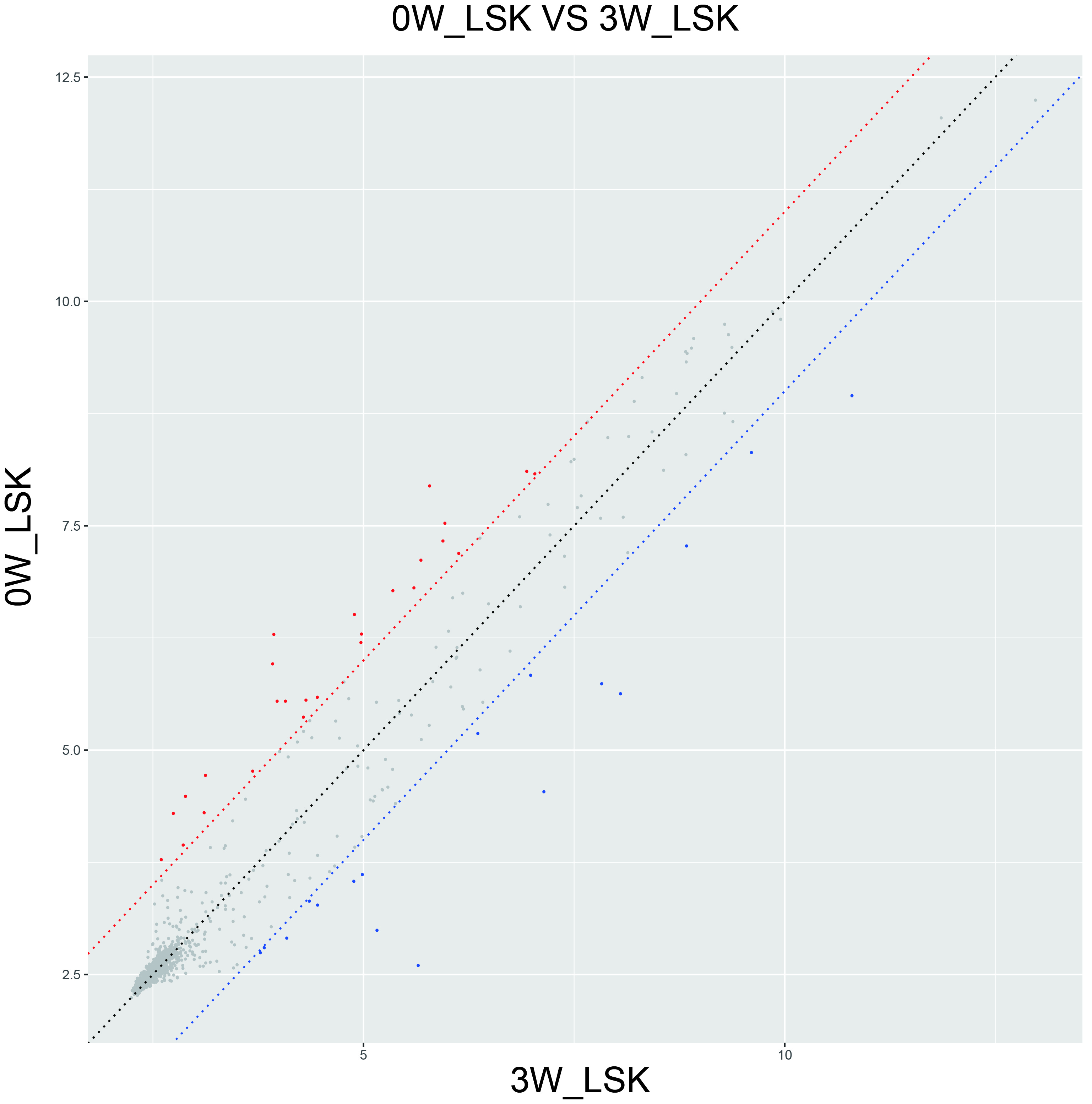


**Figure S2. A comparison of signal in miRNA microarray shown by Scatter plots.** Scatter plots showing a comparison of normalized signal in miRNA microarray between 0W_LSK (y axis) and 3W_LSK (x axis). Red dots and blue dots represent the genes significantly altered after BA expression was induced (fold change ≥ 2 or ≤0.5, at least in one sample, the signal of the probe was significantly different from the background signal).


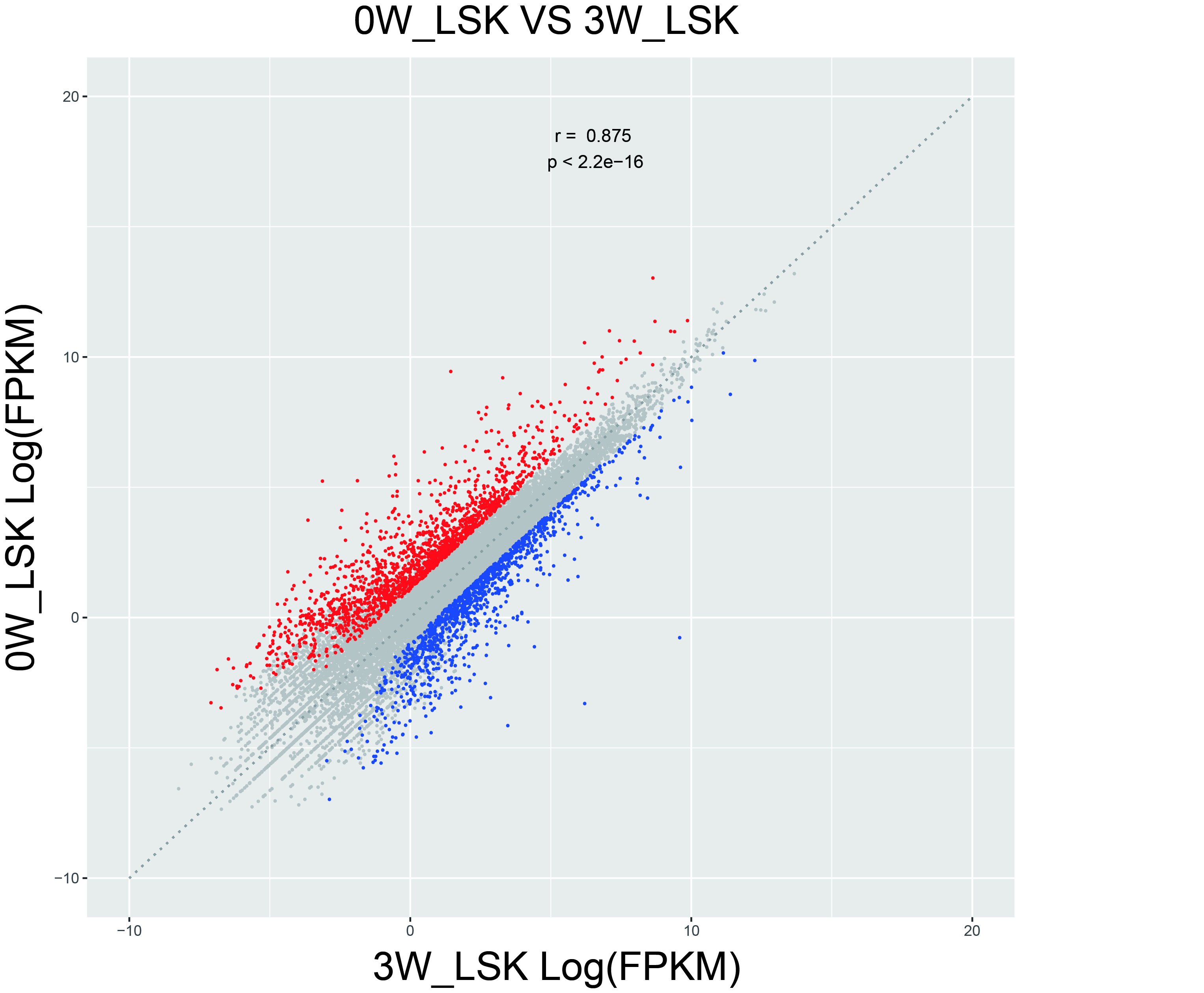


**Figure S3. A comparison of read counts in RNA-seq shown by Scatter plots.**  Scatter plots showing a comparison of read counts in RNA-seq between 0W_LSK (y axis) and 3W_LSK (x axis) with logarithmic scales. Red dots and blue dots represent the genes significantly altered after BA expression was induced (fold change ≥ 2 or ≤0.5, q-value < 0.05).

**
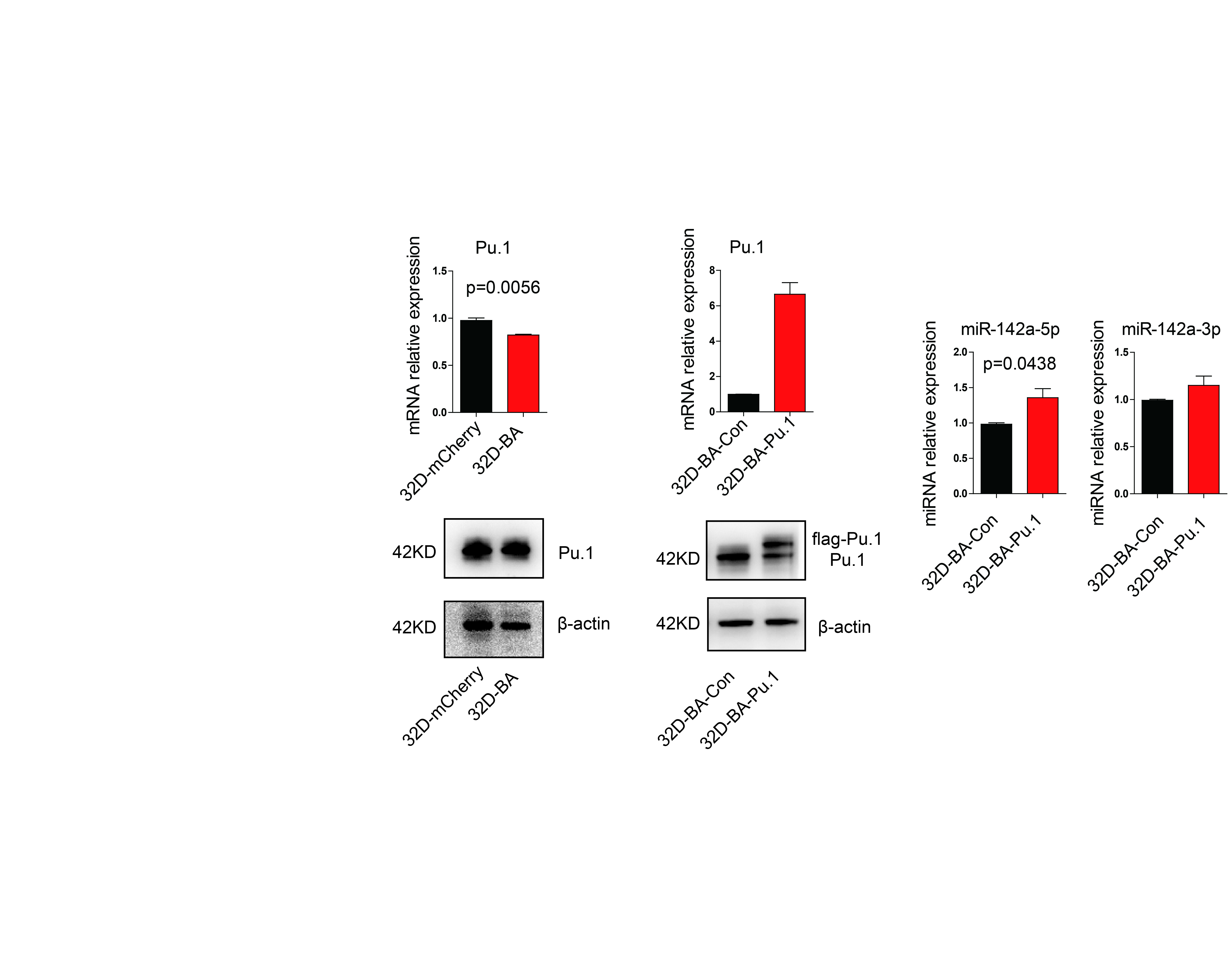
**

B

C

A

**Figure S4. Pu.1 slightly affected miR-142a expression in 32D-BA cells**. **(A)** A slight decrease in both mRNA and protein level of Pu.1 shown in 32D-BA compared with 32D cells. (B) Validation of enforced Pu.1 expression by qRT-PCR and western blot. (C) Pu.1 overexpression induced a slight increase in miR-142a-5p but not miR-142a-3p expression. P value was assessed by the student’s unpaired t-test.

**Table S1. List of upregulated and downregulated miRNAs**

Filter criteria: fold change≥1.5 or ≤0.67. At least in one sample, the signal of the probe is significantly different from the background.

**Table S2. List of top 100 upregulated and downregulated genes**

3122 differentially expressed genes were identified (1196 upregulated, 1926 downregulated, fold change≥2 or ≤0.5). The top 100 upregulated and downregulated genes were showed in this table.
